# Impact of chronic hepatitis C on mortality in cirrhotic patients admitted to intensive-care unit

**DOI:** 10.1186/s12879-016-1448-8

**Published:** 2016-03-12

**Authors:** Alejandro Álvaro-Meca, María A. Jiménez-Sousa, Alexandre Boyer, José Medrano, Holger Reulen, Thomas Kneib, Salvador Resino

**Affiliations:** Departamento de Medicina Preventiva y Salud Pública, Facultad de Ciencias de la Salud, Universidad Rey Juan Carlos, Alcorcón, Madrid Spain; Unidad de Infección Viral e Inmunidad, Centro Nacional de Microbiología, Instituto de Salud Carlos III, Majadahonda, Madrid Spain; Université de Bordeaux, INSERM U657, Pharmaco-épidémiologie et évaluation de l’impact des produits de santé sur les populations, F-33000 Bordeaux cedex, France; Departamento de Medicina, Universidad del País Vasco UPV/EHU, Vitoria-Gasteiz, Spain; Servicio de Urgencias, Hospital Universitario de Araba, Vitoria-Gasteiz, Spain; Chair of Statistics, University of Goettingen, 37073 Göttingen, Germany; Centro Nacional de Microbiología, Instituto de Salud Carlos III (Campus Majadahonda), Carretera Majadahonda- Pozuelo, Km 2.2, 28220 Majadahonda, Madrid Spain

**Keywords:** Critical care, Survival, Chronic hepatitis C, Severe sepsis, HCV

## Abstract

**Background:**

Cirrhosis and severe sepsis are factors associated with increased mortality in intensive care unit (ICU), but chronic hepatitis C (CHC) has been less studied in ICU. The aim of this study was to analyze the impact of CHC on the mortality of cirrhotic patients admitted to ICU according to severe sepsis and decompensated cirrhosis.

**Methods:**

We carried out a retrospective study based on CHC-cirrhotic patients (CHC-group) admitted to ICU (*n* = 1138) and recorded in the Spanish Minimum Basic Data Set (2005–2010). A control-group (randomly selected cirrhotic patients without HIV, HBV, or HCV infections) was also included (*n* = 4127). The primary outcome variable was ICU mortality. The cumulative mortality rate on days 7, 30, and 90 in patients admitted to the ICUs was calculated by dividing the number of deaths by the number of patients admitted to the ICU. The adjusted hazard ratio (aHR) for death in the ICU was estimated through a semi-parametric Bayesian model of competing risk.

**Results:**

The CHC-group had a higher cumulative incidence of severe sepsis than the control-group in compensated cirrhosis (37.4 vs. 31.1 %; *p* = 0.024), but no differences between the CHC-group and the control-group in decompensated cirrhosis were found. Moreover, a higher cumulative incidence of severe sepsis was associated with decompensated cirrhosis compared to compensated cirrhosis in the control-group (40.1 vs. 31.1 %; *p* < 0.001) whereas this was not observed in the CHC group (38.1 vs. 37.4 %; *p* = 0.872). The CHC-group had higher cumulative mortality than the control-group by days 7 (47 vs. 41.3 %; *p* < 0.001), 30 (78.5 vs. 73.5 %; *p* < 0.001), and 90 (96.3 vs. 95.9 %; *p* < 0.001). In a competitive risk model, the CHC-group had a higher risk of dying if the ICU course was complicated by severe sepsis (adjusted hazard ratio (aHR) = 1.19; *p* = 0.003), but no significant values in patients with absence of severe sepsis were found (aHR = 1.09; *p* = 0.068). When patients were stratified by cirrhosis stage and severe sepsis, CHC patients with compensated cirrhosis had the higher risk of death if they had severe sepsis (aHR = 1.35; *p* = 0.002). Moreover, the survival was low in patients with decompensated cirrhosis and severe sepsis but we did not find significant differences between CHC-group and control-group.

**Conclusions:**

CHC was associated with an increased risk of death in cirrhotic patients admitted to ICUs, particularly in patients with compensated cirrhosis and severe sepsis.

**Electronic supplementary material:**

The online version of this article (doi:10.1186/s12879-016-1448-8) contains supplementary material, which is available to authorized users.

## Background

Hepatitis C virus (HCV) is an important cause of liver disease worldwide and constitutes a major global public-health threat. About 9 million people have chronic hepatitis C (CHC) in the European region (3 % population) and 350,000 deaths occur each year as a result of HCV infections [1]. CHC is associated with an increased risk of hospital admission and mortality due to severe liver disease, cirrhosis, end-stage liver disease, and other conditions [2–4]. This increased mortality rate remains despite the administration of the HCV-specific treatment of peg-interferon/ribavirin [5]. However, new direct antiviral agents against HCV mark the beginning of an extraordinary new era in HCV therapy, which will lead to viral eradication in most if not all CHC patients who undergo treatment [6, 7]. A virological cure improves quality of life and reduces the risk of hepatic decompensation events and liver-related deaths [8].

Hepatitis C is associated with increased mortality as HCV-infected individuals have a higher all-cause and a higher liver-related mortality rate compared to the general population [9]. Cirrhosis is a common comorbid condition that complicates the management of patients admitted to an intensive-care unit (ICU) [10], and is associated with increased mortality [11]. In addition, a significant proportion of patients develop decompensated cirrhosis and extra-hepatic organ dysfunction [12]. Moreover, bacterial infections are very common in cirrhotic patients [13] and severe sepsis is more likely to occur in individuals with severe liver disease [14, 15]. Bacterial infections and sepsis represent the most important causes of progressive liver failure, development of liver-related complications, and increased mortality in cirrhotic patients [12, 15]. This susceptibility to infection is caused, at least in part, by defects in the host’s defense, which manifests as “sepsis-like” immune paralysis with reduced cellular immune function [16, 17].

The aim of this study was to analyze whether cirrhotic patients with CHC have a greater tendency to die in ICU than non-CHC cirrhotic subjects through the use of comprehensive records of the Minimum Basic Data Set (MBDS) in Spain.

## Methods

### Study design and data source

A retrospective study with nationwide population-based diagnoses from the Spanish MBDS was performed. We identified all consecutive cirrhotic patients with a record of CHC (CHC-group) and aged >18 years who were admitted to ICUs in Spanish hospitals between January 1, 2005 and December 31, 2010. A control-group of cirrhotic patients without CHC was selected at a proportion of 4:1 with regards to the CHC-group. This control-group contained patients aged >18 years and admitted to the ICU with no record of being tested for HCV, HIV, or HBV; they were randomly selected according to the same frequencies of age, gender, trauma and surgical conditions, and comorbidities (see Additional file 1: Supplementary Digital Content (SDC)-Appendix 1-6) as occurred in the CHC-group [18].

Data were obtained from records in the MBDS of the National Surveillance System for Hospital Data in Spain, provided by the Spanish Ministry of Health. The MBDS is a clinical and administrative database containing clinical information recorded at the time of hospital discharge, which has an estimated coverage of total admissions to public hospitals of 97.7 % [19]. The MBDS provides the encrypted patient identification number, gender, date of birth, dates of hospital admission and discharge, medical institutions providing the services, the diagnosis and procedure codes according to the *International Classification of Diseases 9th edn*, *Clinical Modification* (ICD-9-CM), as well as the outcome at discharge [20].

Length of stay was obtained as the difference, in days, between the date of hospital admission and date of discharge or death in the ICU. The day of hospital admission was considered as day 0. Discharge on the same day was considered as a 1-day stay. For patients admitted several times to the ICU, only the first admission (also called the index episode) was analyzed.

The data were treated with full confidentiality according to Spanish legislation. MBDS is regulated by an law, which explains how institutions have to proceed with health-related personal data. Informed consent is not required because personal data are collected for official usage by public administrations. The study was approved by the Research Ethics Committee (Comité de Ética de la Investigación y de Bienestar Animal) of the Instituto de Salud Carlos III (Madrid, Spain).

### Study groups and ICD-9-CM codes selected

We included patients admitted to an ICU and who were coded in the MBDS. Then, we selected all patients with a cirrhosis diagnosis at the time of hospital discharge, both compensated and decompensated. According to MDBS characteristics, we cannot affirm that all patients had a cirrhosis diagnosis at hospitalization day, but practically we may assume that all patients had cirrhosis when they entered the hospital because cirrhosis is developed over years. Patients with liver cancer or with a liver transplant were excluded (see Additional file 1: SDC-Appendix 2).

The ICD-9-CM codes were also used to define viral-infection status (see Additional file 1: SDC-Appendix 3): i) HIV infection (042 or V08); ii) chronic HCV infection (ICD-9-CM codes 070.44, 070.54, 070.7x, or V02.62); or iii) chronic HBV infection (ICD-9-CM codes 070.2x, 070.3x, or V02.61). Next, we established two groups of patients according to their viral status: i) control-group (patients randomly selected without HIV, HBV, or HCV infections); ii) CHC-group (patients exclusively infected with HCV [both HBV and HIV infections were excluded]).

Severe sepsis was defined by the presence of an infection-associated diagnosis and organ dysfunction according to the criteria of Angus et al. [21], using ICD-9-CM codes (see Additional file 1: SDC-Appendix 4 and 5). The MBDS provides the ICD-9-CM codes for the Angus criteria, but not the date of diagnoses: thus, we were unable to calculate the date of onset of severe sepsis, which was recorded simply as present or absent during the hospital stay.

### Factors and outcome variables

The main factors of study were the HCV serostatus (CHC-group vs. control-group) and type of cirrhosis (compensated vs. decompensated). The outcome variables were the onset of severe sepsis and death.

### Statistical analyses

For descriptive analysis, the results are presented as medians and their interquartile ranges for continuous variables; and as absolute numbers and percentages for categorical data. Categorical data and proportions were analyzed using chi-squared test or Fisher’s exact test, as required. Student’s *t*-test or the Mann–Whitney U tests were used to compare continuous variables.

The primary outcome variable was ICU mortality. The cumulative mortality rate on days 7, 30, and 90 in patients admitted to the ICUs was calculated without considering censoring. This rate was estimated by dividing the number of deaths by the number of patients admitted to the ICU. Log-linear modeling for contingency tables was used to estimate the main and interaction (moderator) effects independently.

We also calculated the probability of death, after taking censoring into account, using a semi-parametric model of competing risk to prevent the results being biased [22]. This analysis determined the effect of CHC on the risk of ICU mortality according to the presence of severe sepsis: I.e., a) risk of ICU mortality in patients with severe sepsis; and b) risk of ICU mortality in patients without severe sepsis. The model was adjusted by the following covariates: age, gender, decompensated cirrhosis, Charlson comorbidity index, abuse of alcohol and/or drugs, number of organ failures, and site of infection (see Additional file 1 SDC). This semi-parametric model provided the survival probabilities and adjusted hazard ratios (aHR).

All analyses were performed using the R statistical package, version 3.1.0 (GNU General Public License) [23] and BayesX software version 2.1 (GNU General Public License) [24]. All tests were two-tailed, with *p*-values of <0.05 considered statistically significant.

## Results

### Patients’ characteristics

Table 1 shows the epidemiological and clinical characteristics of cirrhotic patients included in this study: 4127 patients in the control-group and 1138 in the CHC-group. Overall, the epidemiological and clinical characteristics of the two groups of subjects were quite similar. The most clinically significant differences between groups were a lower length of hospital stay (*p* < 0.001), less abuse of alcohol and/or drugs (*p* < 0.001), and a lower Charlson comorbidity index (*p* < 0.001) in the CHC group compared to the control group. Conversely, the CHC group exhibited a higher frequency of decompensated cirrhosis (*p* < 0.001).

**Table 1:** Epidemiological and clinical characteristics of cirrhotic patients admitted into Spanish intensive care units between 2005 and 2010

	Control-group	CHC-group	*p*-value
No.	4127	1138	
Males	2737 (66.3 %)	719 (63.2 %)	0.053
Age (years)	57.2 (56.7; 57.6)	58.2 (57.4; 58.9)	0.030
Length of hospital stay (days)	12.5 (11.9; 13.1)	10.7 (9.9; 11.6)	<0.001
Abuse of alcohol and drugs	1622 (39.3 %)	342 (30.1 %)	<0.001
Charlson comorbidity index	0.91 (0.86; 0.95)	0.75 (0.68; 0.82)	<0.001
Decompensated cirrhosis	2539 (61.5 %)	777 (68.3 %)	<0.001
General comorbidities			
Cardiovascular	3041 (73.7 %)	830 (72.9 %)	0.638
Infectious without hepatitis	1352 (32.8 %)	383 (33.7 %)	0.594
Respiratory	2212 (53.6 %)	597 (52.5 %)	0.517
Gastrointestinal/hepatic	4029 (97.6 %)	1105 (97.1 %)	0.368
Neurological	911 (22.1 %)	263 (23.1 %)	0.482
Cancer	296 (7.2 %)	93 (8.2 %)	0.281
Diabetes	692 (16.8 %)	210 (18.5 %)	0.196

Values are expressed as absolute numbers (percentages) and as the mean (95 % confidence intervals). *P*-values were calculated by the chi-squared test or Student’s *t*-test, as appropriate

*Abbreviation*: *CHC* chronic hepatitis C virus

### Severe sepsis in cirrhotic patients admitted into ICUs

Table 2 shows that severe sepsis in the CHC-group and controls had a similar cumulative incidence (37.9 vs. 36.6 %; *p* = 0.456). Respiratory failure was the most common organ failure and the digestive tract was the most common site of infection in both groups.

**Table 2:** Cumulative incidence of severe sepsis in cirrhotic patients admitted to intensive-care units between 2005 and 2010

	Control-group	CHC-group	*p*-value
Severe sepsis	1511 (36.6 %)	431 (37.9 %)	0.456
Acute organ dysfunction			
Respiratory	1329 (88 %)	365 (84.7 %)	0.963
Cardiovascular	1018 (67.4 %)	283 (65.7 %)	0.920
Renal	1036 (68.6 %)	288 (66.8 %)	0.919
Hematological	469 (31 %)	124 (28.8 %)	0.697
Metabolic	254 (16.8 %)	84 (19.5 %)	0.154
Neurological	222 (14.7 %)	43 (10 %)	0.035
Hepatic	556 (36.8 %)	146 (33.9 %)	0.606
Site of infection			
Respiratory	515 (34.1 %)	157 (36.4 %)	0.259
Digestive	896 (59.3 %)	267 (61.9 %)	0.222
Genitourinary	171 (11.3 %)	46 (10.7 %)	0.946
Central nervous system	16 (1.1 %)	9 (2.1 %)	0.132
Skin, soft tissue, or bone	87 (5.8 %)	33 (7.7 %)	0.141
Circulatory	39 (2.6 %)	8 (1.9 %)	0.555

Values are expressed as absolute numbers (percentages). *P*-values were calculated using the chi-squared test. The sum of sites of infection is greater than the number of patients with severe sepsis because a patient may have more than one site of infection

*Abbreviation*: *CHC* chronic hepatitis C virus

Table 3 shows the cumulative incidence of severe sepsis in cirrhotic patients after stratifying patients by type of cirrhosis (compensated or decompensated). The CHC-group had a higher cumulative incidence of severe sepsis than the control-group in compensated cirrhosis (37.4 vs. 31.1 %; *p* = 0.024), but no differences between the CHC-group and the control-group in decompensated cirrhosis were found. Moreover, a higher cumulative incidence of severe sepsis was associated with decompensated cirrhosis compared to compensated cirrhosis in the control-group (40.1 vs. 31.1 %; *p* < 0.001) whereas this was not observed in the CHC group (38.1 vs. 37.4 %; *p* = 0.872).

**Table 3:** Cumulative incidence of severe sepsis in cirrhotic patients admitted to intensive-care units between 2005 and 2010 according to the absence or presence of decompensated cirrhosis

	All patients	Control-group	CHC-group	*p*-values
Compensated cirrhosis	629 (32.3 %)	494 (31.1 %)	135 (37.4 %)	0.024
Decompensated cirrhosis	1313 (39.6 %)	1017 (40.1 %)	296 (38.1 %)	0.349
*p*-values	<0.001	<0.001	0.872	

Values are expressed as absolute counts (percentages). *P*-values were calculated using the chi-squared test

*Abbreviation*: *CHC* chronic hepatitis C

### Cumulative crude mortality rate in patients admitted to the ICU

**Fig. 1:**
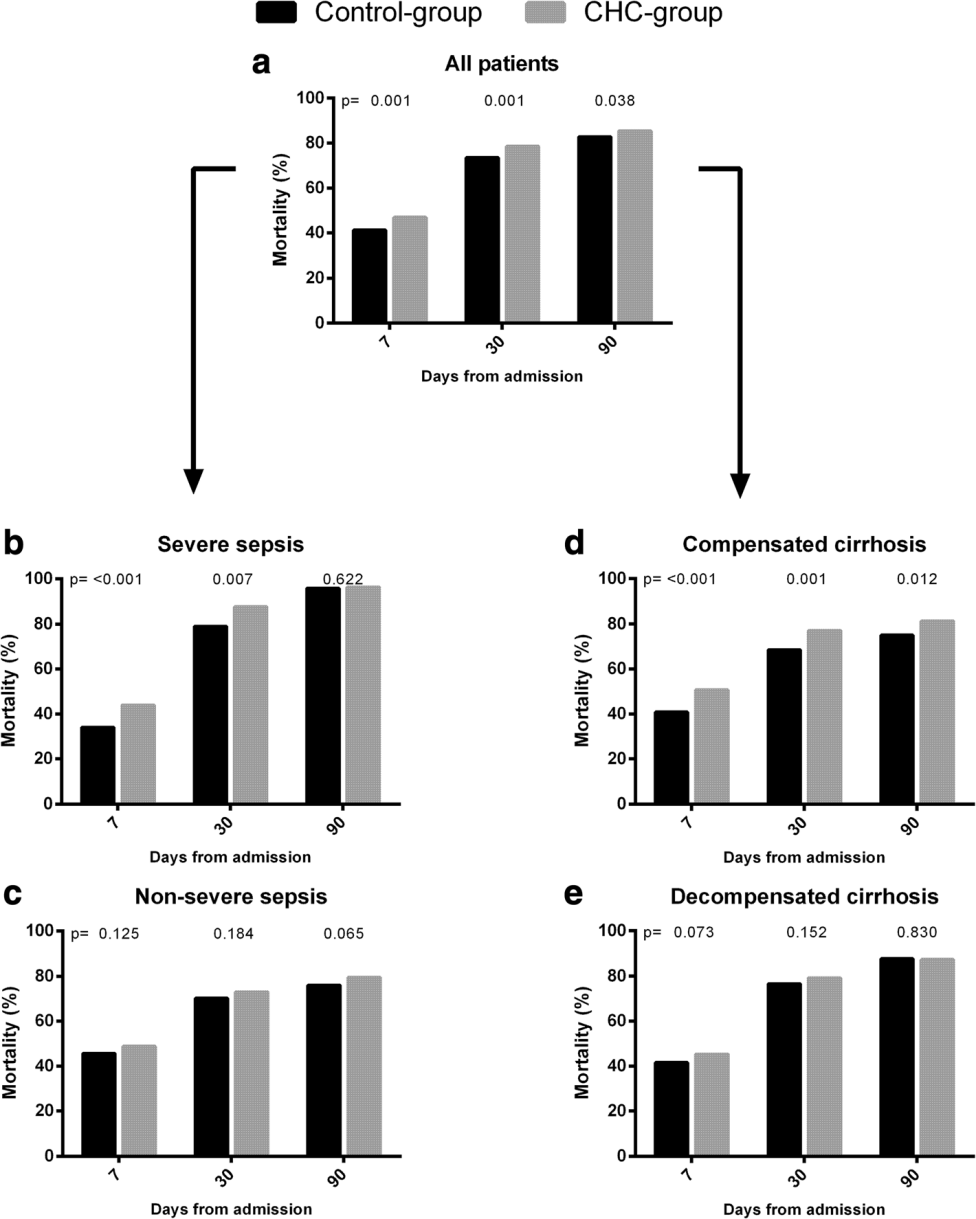
Cumulative crude mortality rates by days 7, 30, and 90 of cirrhotic patients admitted to Spanish ICUs between 2005 and 2010. Values are expressed as absolute counts (percentages). *P*-values were calculated using the chi-squared test. *Abbreviations*: *ICU* intensive-care unit, *CHC* chronic hepatitis C. (**a**) All patients; (**b**), Patients with severe sepsis; (**c**), Patients without severe sepsis; (**d**), Patients with compensated cirrhosis; (**e**), Patients with decompensated cirrhosis

Overall, the CHC-group had a higher ICU mortality rate than the control-group at days 7 (47 vs 41.3 %; *p* = 0.001), 30 (78.5 vs. 73.5 %; *p* = 0.001), and 90 (85.4 vs. 82.8 %; *p* = 0.038) (Fig. 1a). When stratified for severe sepsis, the CHC-group still had higher ICU mortality rates than the control-group at days 7 (43.9 vs. 34 %; *p* < 0.001) and 30 (87.5 vs. 79 %; *p* < 0.001), but no significant differences between the CHC-group and control-group in patients without severe sepsis were found (Fig. 1b and c). When stratified by cirrhosis stage, the CHC-group with compensated cirrhosis had a higher rate of ICU mortality than the control-group at days 7 (50.7 vs. 40.9 %; *p* = 0.001), 30 (77 vs. 68.5 %; *p* = 0.002), and 90 (81.4 vs. 75 %; *p* = 0.012), but no significant differences between the CHC-group and the control-group in patients with decompensated cirrhosis were found (Fig. 1d and e).

### Estimated risk of death in patients admitted to the ICU

**Fig. 2:**
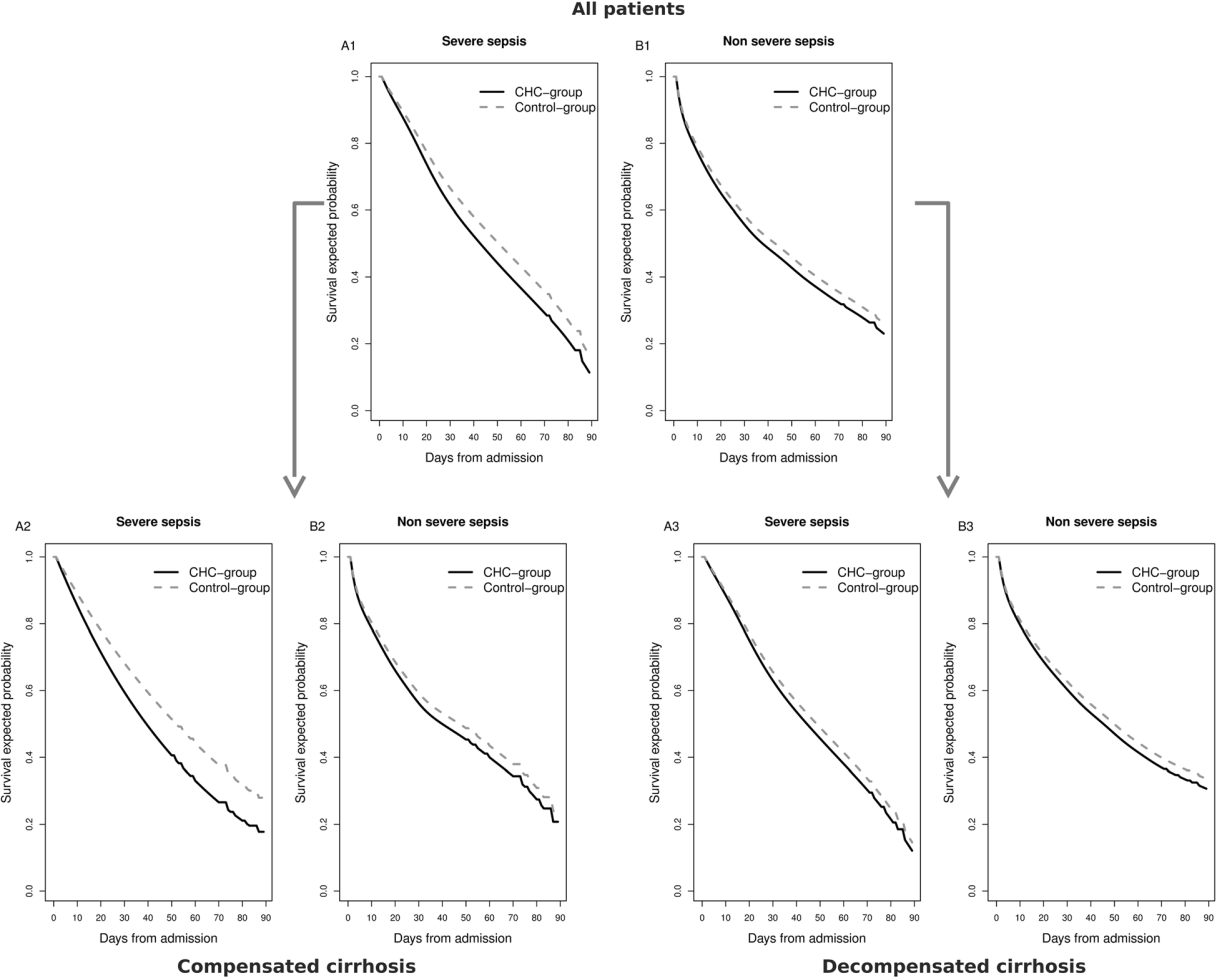
Estimated survival of cirrhotic patients admitted to intensive-care units (ICU) between 2005 and 2010. Survival functions were calculated using a competing risk model (see Statistical analyses section). *Abbreviations*: *CHC* chronic hepatitis C. (**a**) Patients with severe sepsis; (**b**), Patients without severe sepsis; (A1), Patients with severe sepsis and compensated cirrhosis; (A2), Patients without severe sepsis and compensated cirrhosis; (B1), Patients with severe sepsis and decompensated cirrhosis; (B2), Patients without severe sepsis and decompensated cirrhosis

**Fig. 3:**
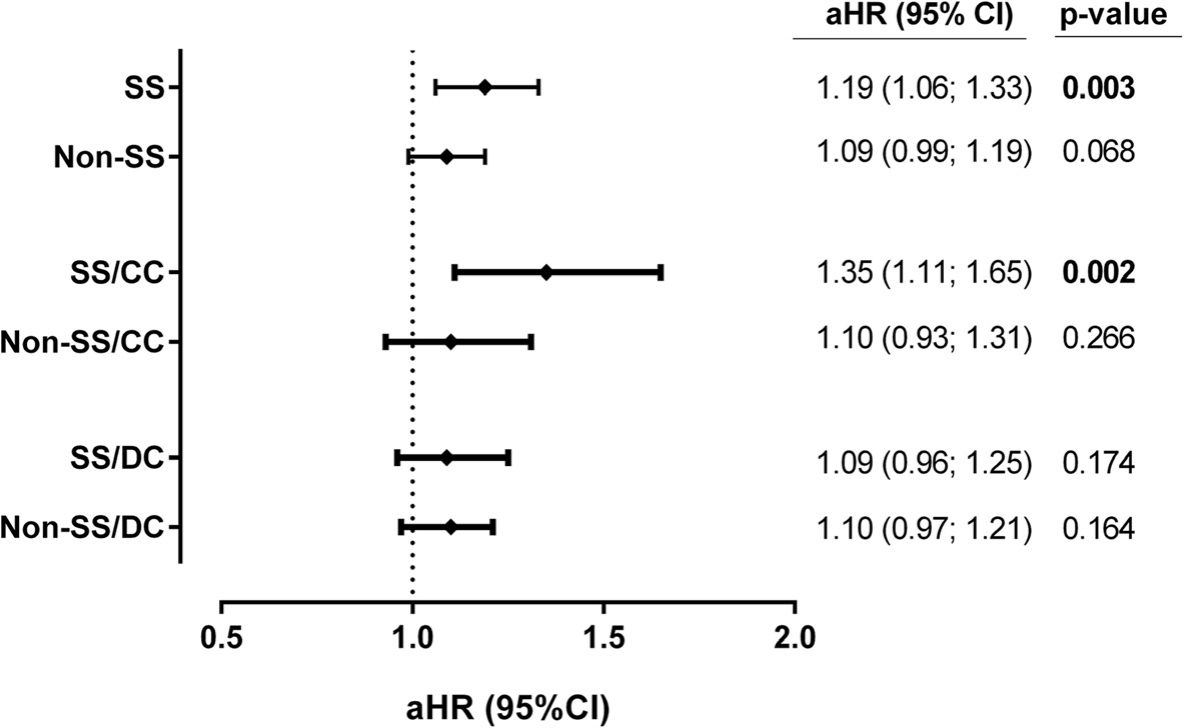
Adjusted risk of death of cirrhotic patients with chronic hepatitis C admitted to intensive-care units (ICU) between 2005 and 2010, compared to a control-group. Adjusted hazard ratios were calculated by a competing risk model (see Statistical analyses section). *Abbreviations*: *aHR* adjusted hazard ratio, *95 % CI* 95 % confidence interval, *SS* severe sepsis, *Non-SS* non-severe sepsis, *CC* compensated cirrhosis, *DC* decompensated cirrhosis

The CHC-group had lower estimated survival in the ICUs compared to the control-group, especially in patients with severe sepsis (Fig. 2a1). Thus, the CHC-group had a higher risk of dying if the ICU course was complicated by severe sepsis (aHR = 1.19; *p* = 0.003), but no significant values in patients with absence of severe sepsis were found (aHR = 1.09; *p* = 0.068) (Fig. 3). When patients were stratified by cirrhosis stage and severe sepsis, the CHC-group had lower survival compared to the control-group in patients with compensated cirrhosis and severe sepsis (Fig. 2A2). No significant differences between the CHC-group and the control-group were found in the other strata (Fig. 2b2-a3-b3). Thus, CHC patients with compensated cirrhosis had the higher risk of death if they had severe sepsis (aHR = 1.35; *p* = 0.002) (Fig. 3). Moreover, the survival was low in patients with decompensated cirrhosis and severe sepsis but we did not find significant differences between the CHC-group and the control-group (Fig. 2a3). Thus, patients in the CHC-group with decompensated cirrhosis had not a significant risk of dying compared with the control-group, independently of the onset of severe sepsis (Fig. 3).

## Discussion

Cirrhosis and mortality have been widely studied in individuals admitted to ICUs [10, 11, 15, 25, 26]; but to our knowledge, no previous studies have investigated the influence of CHC on ICU mortality among cirrhotic patients. Our manuscript provides a nationwide view of mortality rates of CHC-cirrhotic patients admitted into ICUs in Spain. In this study, the major findings were: 1) patients with CHC had a higher cumulative incidence of severe sepsis compared to patients without CHC in the subgroup of compensated cirrhosis, whereas the cumulative incidence of severe sepsis did not differ among patients with decompensated cirrhosis; 2) cirrhotic patients with CHC (CHC-group) had a greater tendency to die in ICU than non-CHC subjects (control-group); 3) The influence of CHC was observed on patients with compensated cirrhosis and severe sepsis, whereas CHC seemed to have no impact on mortality in patients with decompensated cirrhosis, independently of the onset of severe sepsis. Note that several baseline clinical characteristics showed statistically significant differences between groups, but these variables were included in the model to adjust the competitive risks.

There is controversial about whether patients with cirrhosis may benefit from the ICU management. In our study, the mortality was extremely high (merely 80 %) in comparison to data reported in the review of Saliba et al. [18]. There also are several important series published during the last 5 years with a mortality about 50 % [59 % Das et al. [27], 70.1 % Galbois et al. [28], 58.8 % Pan et al. [29], 68.1 % Bao et al. [30], 60 % McPhail et al. [31]]. These differences in death rates could be due to the designs of the studies used to obtain the data, which were very different, especially in our study carried out with data of Spanish MBDS from 2005 to 2010 and with a higher number of patients.

A systemic response to infection is more intense in the presence of cirrhosis, which translates into a greater risk of developing severe sepsis [14]. Thus, cirrhotic patients admitted into ICUs, apart from having a higher prevalence of infection than non-cirrhotic patients, have a higher rate of sepsis and death [13]. Furthermore, decompensated cirrhosis predisposes to delayed intestinal transit, increasing intestinal permeability and facilitating bacterial translocation from the gastro-intestinal lumen to the systemic circulation. This is accompanied by cirrhosis-associated immune dysfunction, which encourages systemic inflammation [32]. In our study, cirrhotic patients with and without CHC had similar rates of severe sepsis. However, when patients were stratified by the presence or absence of decompensated cirrhosis, the CHC-group with compensated cirrhosis had a higher cumulative incidence of severe sepsis than expected; which suggests that, in patients with compensated cirrhosis, susceptibility to severe sepsis may be increased by CHC.

The severity of infection is higher in cirrhotic patients than in non-cirrhotic patients [12, 15]. In addition, infections are increasingly recognized as a major trigger of systemic inflammation and organ failure in decompensated cirrhosis, leading to four-fold increased mortality rates [33]. In this setting, bacterial infections and sepsis are recognized as a distinct stage in the natural progression of chronic liver disease as they accelerate organ failure and contribute to the high mortality observed in decompensated cirrhosis [32]. In our study, as expected, cirrhotic patients with severe sepsis had a higher risk of ICU mortality than patients without severe sepsis; and patients with decompensated cirrhosis had a higher risk of ICU mortality compared to patients with compensated cirrhosis, independently of CHC status. During cirrhosis, sepsis is accompanied by a markedly imbalanced cytokine response with increased tissue damage and inflammation [14]. Moreover, patients with end-stage liver disease have enhanced intestinal permeability and translocation of bacteria and their products, imbalanced immune response, and aggravated intrahepatic microcirculatory dysfunction, which causes accumulation of toxins and immune dysfunction that may perpetuate end-stage organ dysfunction [34], and may explain the higher mortality rates [35].

As discussed above, HCV infection is a major cause of cirrhosis [36], and cirrhotic patients in ICUs have higher risks of sepsis and death, particularly in end-stage liver disease [15]. In our study, CHC appears to be a key factor that increases the risk of death in compensated cirrhotic patients with severe sepsis in ICUs, but the effect of CHC status was not maintained in other patients with compensated cirrhosis/non-severe sepsis or patients with decompensated cirrhosis (with or without severe sepsis). However, we have no clear explanation for this effect observed exclusively in patients with compensated cirrhosis and severe sepsis. On the one hand, CHC-associated comorbidities could promote negative clinical outcomes among cirrhotic patients, thus causing a significant proportion of the mortalities of CHC patients [37]. On the other hand, persistent HCV infection is the result of a series of connected events that culminate in diminished immunity and the inability to eliminate HCV infection [38]. This immune dysfunction is accompanied by inflammation and immune activation during CHC [38], which could be crucial in the prognosis of CHC-cirrhotic patients. Thus, it could be possible that CHC increases the immune dysfunction that occurs with severe sepsis, worsening the prognosis of cirrhotic patients. In this context, the effect of CHC could be diluted in the presence of decompensated cirrhosis in patients with a more advanced stage of disease, thus resulting in a worse prognosis, whereas the effect of CHC could be clearly observed in patients with compensated cirrhosis, as observed in our study.

Several points should be taken into account for the correct interpretation of our results (Limitations of the study):

Firstly, this study was retrospective and the acquisition of some clinical data related to HCV infection (viral status, patients who received prior treatment by antivirals, etc.) and the ICU (community acquired or nosocomial nature of the sepsis, or prognostic scores such as Child-Pugh, MELD, SOFA or CLIF-SOFA score) were unavailable from the MBDS records. Furthermore, we do not know the reason for admission of these patients; therefore, the prognostic could have been different if the patients were admitted for gastrointestinal bleeding, or for severe sepsis or septic shock. Neither do we know if these patients were admitted due to severe sepsis or acquired the sepsis in the ICU prior to death.

Secondly, the time until death or discharge was calculated from entry into the hospital rather than the ICU because the date of ICU admission was not recorded in the MBDS. A time bias could have occurred [37], since some patients may have been admitted directly into an ICU whereas others may have had a period of time between hospital admission and entry into an ICU, and this time period was included in the observation time. However, this theoretical bias should be well-balanced between the groups, and should only affect survival time.

Thirdly, due to the use of the administrative databases, was the inaccuracy in differentiating the etiologies of the diseases and the reporting of organ dysfunction, which could have engendered a confusion bias. In this context, grouping of ICD-9-CM codes into comorbidities, organ dysfunction, and site of infection (Additional file 1: SDC-Appendixes 1–6) may have been the best approach to solve this issue. On the other hand, MBDS has already proven its usefulness in the previous assessments of outcomes among patients admitted to ICU [39, 40]. This database has advantages being a national clinical administrative database, which allows analyze the trends in important public health issues.

## Conclusions

CHC was associated with an increased risk of death in cirrhotic patients admitted to ICUs, particularly in patients with compensated cirrhosis and severe sepsis.

## Additional file

Additional file 1: Appendix 1. International Classification of Diseases, 9th Revision, Clinical Modification (ICD-9-CM) codes for comorbid diseases. Appendix 2. International Classification of Diseases, 9th Revision, Clinical Modification (ICD-9-CM) codes for CHC related diagnoses and abuse of alcohol and drugs. Appendix 3. International Classification of Diseases, 9th Revision, Clinical Modification (ICD-9-CM) codes for HIV, HCV and HBV status. Appendix 4. International Classification of Diseases, 9th Revision, Clinical Modification (ICD-9-CM) codes for acute organ dysfunction. Appendix 5. International Classification of Diseases, 9th Revision, Clinical Modification (ICD-9-CM) codes for bacterial and fungal infections. Appendix 6. International Classification of Diseases, 9th Revision, Clinical Modification (ICD-9-CM) coding algorithms for Charlson comorbidities. (DOC 219 kb)

## Abbreviations

HCV: hepatitis C virus
CHC: chronic hepatitis C
ICU: intensive care unit
MBDS: minimum basic data set
ICD-9-CM: International Classification of Diseases, 9th ed, Clinical Modification
SDC: supplementary digital content
CHC-group: patients exclusively infected with HCV
Control-group: patients randomly selected without HIV, HBV, or HCV infections
aHR: adjusted hazard ratio
