# Supplementary material for: Impact of chronic hepatitis C on mortality in cirrhotic patients admitted to intensive-care unit
Source: BMC Infect Dis. 2016 Mar 12;16:122. doi: 10.1186/s12879-016-1448-8 (PMC4793506; doi:10.1186/s12879-016-1448-8)
Supplement: Additional file 1: — Appendix 1. International Classification of Diseases, 9th Revision, Clinical Modification (ICD-9-CM) codes for comorbid diseases. Appendix 2. International Classification of Diseases, 9th Revision, Clinical Modification (ICD-9-CM) codes for CHC related diagnoses and abuse of alcohol and drugs. Appendix 3. International Classification of Diseases, 9th Revision, Clinical Modification (ICD-9-CM) codes for HIV, HCV and HBV status. Appendix 4. International Classification of Diseases, 9th Revision, Clinical Modification (ICD-9-CM) codes for acute organ dysfunction. Appendix 5. International Classification of Diseases, 9th Revision, Clinical Modification (ICD-9-CM) codes for bacterial and fungal infections. Appendix 6. International Classification of Diseases, 9th Revision, Clinical Modification (ICD-9-CM) coding algorithms for Charlson comorbidities. (DOC 219 kb) [file 12879_2016_1448_MOESM1_ESM.doc]

**Additional file 1 (Appendix 1-6)**

**Appendix 1.** *International Classification of Diseases, 9th Revision, Clinical Modification* (ICD-9-CM) codes for comorbid diseases. Adapted from Cooke et al. .

| **Disease** | **ICD-9 CM Code Definition** |
| --- | --- |
| Cardiovascular | 390* to 429*, 440* to 459* |
| Infectious | 001* to139* |
| Respiratory | 460* to 519* |
| Gastrointestinal/Hepatic | 520* to 579* |
| Neurologic | 320* to 389*, 430* to 438*, 800* to 959*, E800 to E848*, |
| Trauma | E880* to E929*, E950 to E999* |
| Cancer | 140* to 239* |
| Diabetes mellitus | 250* |

ICD-9-CM, International Classification of Diseases, Ninth Revision, Clinical Modification.

* represents inclusion of all fourth and/or fifth digit of the respective ICD-9 CM codes

**Appendix 2.** *International Classification of Diseases, 9th Revision, Clinical Modification* (ICD-9-CM) codes for CHC related diagnoses and abuse of alcohol and drugs.

| **CHC-related diagnoses** | | **ICD-9-CM codes** |
| --- | --- | --- |
| *Chronic liver disease and cirrhosis* | |  |
|  | Chronic liver disease and cirrhosis | 571.xx |
| *Decompensated cirrhosis* | |  |
|  | Awaiting organ transplant status | V49.83 |
|  | Chronic HCV with hepatic coma | 070.44 |
|  | Unspecified hepatitis with hepatic coma | 070.71 |
|  | Encephalopathy not otherwise specified | 348.3x |
|  | Esophageal varices in diseases classified elsewhere with or without bleeding | 456.0, 456.1, 456.2x |
|  | Hepatic encephalopathy | 572.2 |
|  | Portal hypertension | 572.3 |
|  | Hepatorenal syndrome | 572.4 |
|  | Other sequelae of chronic liver disease | 572.8 |
|  | Jaundice | 782.4 |
|  | Ascites | 789.5 |
| *Liver cancer* | |  |
|  | Malignant neoplasm of liver and intrahepatic bile duct | 155 |
| *Liver transplant* | |  |
|  | Liver transplant | V42.7 |
|  | Complications of transplanted liver | 996.82 |
|  | Liver transplant (auxiliary/other) | 50.5x |
| **Abuse of alcohol and drugs** | |  |
|  | Alcohol dependence syndrome | 303.xx |
|  | Drug dependence | 304.xx |
|  | Nondependent abuse of drugs | 305.xx |
|  | Drug Psychoses | 292.xx |
|  | Alcoholic Psychoses | 291.xx |

**Appendix 3.** *International Classification of Diseases, 9th Revision, Clinical Modification* (ICD-9-CM) codes for HIV, HCV and HBV status.

| **Viral infection diagnoses** | | **ICD-9-CM codes** |
| --- | --- | --- |
| *HIV infection* | |  |
|  | Human immunodeficiency virus [HIV] disease | 042 |
|  | Asymptomatic human immunodeficiency virus [HIV] infection status | V08 |
|  |  |  |
| *HCV infection* | |  |
|  | Chronic hepatitis C with hepatic coma | 070.44 |
|  | Chronic hepatitis C without mention of hepatic coma | 070.54 |
|  | Unspecified viral hepatitis c | 070.7x |
|  | Hepatitis C carrier | V02.62 |
|  |  |  |
| *HBV infection* | |  |
|  | Viral hepatitis b with hepatic coma | 070.2x |
|  | Viral hepatitis b without mention of hepatic coma | 070.3x |
|  | Hepatitis B carrier | V02.61 |

**Appendix 4.** *International Classification of Diseases, 9th Revision, Clinical Modification* (ICD-9-CM) codes for acute organ dysfunction. Adapted from Shen et al. , Bateman et al. , and Angus et al. .

| **Organ System** | **ICD-9-CM Code** | **ICD-9-CM Code Description** |
| --- | --- | --- |
| Cardiovascular | 427.5 | Cardiac arrest |
|  | 458.0 | Orthostatic hypotension |
|  | 458.8 | Other specified hypotension |
|  | 458.9 | Hypotension, unspecified |
|  | 785.5 | Shock without mention of trauma |
|  | 796.3 | Hypotension, transient |
| Hematologic | 286.2 | Disseminated intravascular coagulation |
|  | 286.6 | Defibrination syndrome |
|  | 286.9 | Other and unspecified coagulation defects |
|  | 287.4 | Secondary thrombocytopenia |
|  | 287.5 | Thrombocytopenia, unspecified |
|  | 790.92 | Abnormal coagulation profile |
| Hepatic | 570 | Acute and subacute necrosis of liver |
|  | 572.2 | Hepatic encephalopathy |
|  | 573.3 | Hepatitis (septic & not elsewhere classified) |
|  | 573.4 | Hepatic infarction |
| Neurologic | 293 | Transient organic psychosis |
|  | 348.1 | Anoxic brain damage |
|  | 348.3 | Encephalopathy, acute |
|  | 780.01 | Coma |
|  | 780.09 | Altered consciousness, unspecified |
|  | 89.14 | Electroencephalography |
| Renal | 580.x | Acute glomerulonephritis |
|  | 584.x | Acute renal failure |
|  | 586 | Renal shutdown, renal failure unspecified |
|  | 39.95 | Hemodialysis |
| Respiratory | 518.5 | Pulmonary insufficiency following trauma and surgery |
|  | 518.8 | Respiratory failure |
|  | 786.03 | Apnea |
|  | 799.1 | Respiratory arrest |
|  | 786.09 | Respiratory insufficiency |
|  | 96.7 | Other Continuous Invasive Mechanical Ventilation |
|  | 96.04 | Endotracheal intubation (emergency procedure) |
|  | 93.90 | Continuous positive airway pressureº |
| Metabolic | 276.2 | Acidosis, metabolic or lactic |

ICD-9-CM, International Classification of Diseases, Ninth Revision, Clinical Modification.

**Appendix 5.** *International Classification of Diseases, 9th Revision, Clinical Modification* (ICD-9-CM) codes for bacterial and fungal infections. Adapted from Angus et al. and Wang et al. .

| **ICD-9-CM Code** | **ICD-9-CM Code Description** |
| --- | --- |
| 001 | Cholera |
| 002 | Typhoid/paratyphoid fever |
| 003 | Other salmonella infection |
| 004 | Shigellosis |
| 005 | Other food poisoning |
| 008 | Intestinal infections due to other organisms |
| 009 | Ill-defined intestinal infection |
| 013 | Central nervous system tuberculosis |
| 014 | Tuberculosis of intestines peritoneum and mesenteric glands |
| 015 | Tuberculosis of bones and joints |
| 016 | Tuberculosis of genitourinary system |
| 017 | Tuberculosis of other organs |
| 018 | Miliary tuberculosis |
| 020 | Plague |
| 021 | Tularemia |
| 022 | Anthrax |
| 023 | Brucellosis |
| 024 | Glanders |
| 025 | Melioidosis |
| 026 | Rat-bite fever |
| 027 | Other bacterial zoonoses |
| 030 | Leprosy |
| 031 | Diseases due to other mycobacteria |
| 032 | Diphtheria |
| 033 | Whooping cough |
| 034 | Streptococcal throat/scarlet fever |
| 035 | Erysipelas |
| 036 | Meningococcal infection |
| 037 | Tetanus |
| 038 | Septicemia |
| 039 | Actinomycotic infections |
| 040 | Other bacterial diseases |
| 041 | Bacterial infection in other diseases not specified |
| 090 | Congenital syphilis |
| 091 | Early syphilis symptomatic |
| 092 | Early syphilis latent |
| 093 | Cardiovascular syphilis |
| 094 | Neurosyphilis |
| 095 | Other forms of late syphilis with symptoms |
| 096 | Late syphilis, latent |
| 097 | Other and unspecified syphilis |
| 098 | Gonococcal infections |
| 100 | Leptospirosis |
| 101 | Vincent’s angina |
| 102 | Yaws |
| 103 | Pinta |
| 104 | Other spirochetal infection |
| 110 | Dermatophytosis |
| 111 | Dermatomycosis other and unspecified |
| 112 | Candidiasis |
| 114 | Coccidioidomycosis |
| 115 | Histoplasmosis |
| 116 | Blastomycotic infection |
| 117 | Other mycoses |
| 118 | Opportunistic mycoses |
| 320 | Bacterial meningitis |
| 321 | Meningitis due to other organisms |
| 324 | Central nervous system abcess |
| 325 | Phlebitis of intracranial sinus |
| 360.0 | Purulent endophthalmitis |
| 376.0 | Acute inflammation of orbit |
| 380.14 | Malignant otitis externa |
| 383.0 | Acute mastoiditis |
| 420 | Acute pericarditis |
| 421 | Acute or subacute endocarditis |
| 451 | Phlebitis and thrombophlebitis |
| 461 | Acute sinusitis |
| 462 | Acute pharyngitis |
| 463 | Acute tonsillitis |
| 464 | Acute laryngitis/tracheitis |
| 465 | Acute upper respiratory infection of multiple sites/not otherwise specified |
| 475 | Peritonsillar abscess |
| 481 | Pneumococcal pneumonia |
| 482 | Other bacterial pneumonia |
| 485 | Bronchopneumonia with organism not otherwise specified |
| 486 | Pneumonia, organism not otherwise specified |
| 491.21 | Obstructive chronic bronchitis with (acute) exacerbation |
| 491.22 | Obstructive chronic bronchitis with acute bronchitis |
| 494 | Bronchiectasis |
| 510 | Empyema |
| 513 | Abscess of lung and mediastinum |
| 522.5 | Periapical abscess without sinus |
| 522.7 | Periapical abscess with sinus |
| 526.4 | Inflammatory conditions of the jaw |
| 527.3 | Abscess of the salivary glands |
| 528.3 | Cellulitis and abscess of oral soft tissue |
| 540 | Acute appendicitis |
| 541 | Appendicitis not otherwise specified |
| 542 | Other appendicitis |
| 562.01 | Diverticulitis of the small intestine without hemorrhage |
| 562.03 | Diverticulitis of the small intestine with hemorrhage |
| 562.11 | Diverticulitis of colon without hemorrhage |
| 562.13 | Diverticulitis of colon with hemorrhage |
| 566 | Abscess of the anal and rectal regions |
| 567 | Peritonitis |
| 569.5 | Intestinal abscess |
| 569.61 | Infection of colostomy or enterostomy |
| 569.83 | Perforation of intestine |
| 572.0 | Abscess of liver |
| 572.1 | Portal pyemia |
| 575.0 | Acute cholecystitis |
| 590 | Kidney infection |
| 599.0 | Urinary tract infection not otherwise specified |
| 601 | Prostatic inflammation |
| 604 | Orchitis and epididymitis |
| 614 | Female pelvic inflammation disease |
| 615 | Inflammatory diseases of uterus except cervix |
| 616.3 | Abcess of Bartholin’s gland |
| 616.4 | Other abcess of vulva |
| 634.0 | Spontaneous abortion, complicated by genital tract and pelvic infection |
| 635.0 | Legally induced abortion, complicated by genital tract and pevic infection |
| 636.0 | Illegally induced abortion, complicated by genital tract and pelvic infection |
| 637.0 | Unspecified abortion, complicated by genital tract and pelvic infection |
| 638.0 | Failed attempted abortion, complicated by genital tract and pelvic infection |
| 639.0 | Complications following abortion and ectopic and molar pregnancies,genital and pelvic infection |
| 646.6 | Infections of genitourinary tract in pregnancy |
| 658.4 | Infection of amniotic cavity |
| 670 | Major puerperal infection |
| 675.1 | Abscess of breast |
| 681 | Cellulitis, finger/toe |
| 682 | Other cellulitis or abscess |
| 683 | Acute lymphadenitis |
| 685.0 | Pilonidal cyst, with abscess |
| 686 | Other local skin infection |
| 711.0 | Pyogenic arthritis |
| 728.86 | Necrotizing fasciitis |
| 730 | Osteomyelitis |
| 790.7 | Bacteremia |
| 958.3 | Posttraumatic wound infection, not elsewhere classified |
| 996.6 | Infection or inflammation of device/graft |
| 998.5 | Postoperative infection |
| 999.3 | Infectious complication of medical care not otherwise classified |

**Appendix 6.** *International Classification of Diseases, 9th Revision, Clinical Modification* (ICD-9-CM) coding algorithms for Charlson comorbidities. Adapted from Quan et al. .

| **Comorbidities** | **ICD-9-CM** |
| --- | --- |
| Myocardial infarction | 410.x, 412.x |
| Congestive heart failure | 428.x |
| Peripheral vascular disease | 443.9, 441.x, 785.4, V43.4, Procedure 38.48 |
| Cerebrovascular disease | 430.x–438.x |
| Dementia | 290.x |
| Chronic pulmonary disease | 490.x–505.x, 506.4 |
| Rheumatic disease | 710.0, 710.1, 710.4, 714.0–714.2, 714.81, 725.x |
| Peptic ulcer disease | 531.x–534.x |
| Mild liver disease | 571.2, 571.4–571.6 |
| Diabetes without chronic complication | 250.0–250.3, 250.7 |
| Diabetes with chronic complication | 250.4–250.6 |
| Hemiplegia or paraplegia | 344.1, 342.x |
| Renal disease | 582.x, 583–583.7, 585.x, 586.x, 588.x |
| Any malignancy, including lymphoma and leukemia, except malignant neoplasm of skin | 140.x–172.x, 174.x.–195.8, 200.x–208.x |
| Moderate or severe liver disease | 456.0–456.21, 572.2–572.8 |
| Metastatic solid tumor | 196.x–199.1 |
| AIDS/HIV | 042.x–044.x |

**REFERENCES**

1. Cooke CR, Hotchkin DL, Engelberg RA, Rubinson L, Curtis JR. Predictors of time to death after terminal withdrawal of mechanical ventilation in the ICU. Chest **2010** Aug;138(2):289-97.

2. Shen HN, Lu CL, Yang HH. Epidemiologic trend of severe sepsis in Taiwan from 1997 through 2006. Chest **2010** Aug;138(2):298-304.

3. Bateman BT, Schmidt U, Berman MF, Bittner EA. Temporal trends in the epidemiology of severe postoperative sepsis after elective surgery: a large, nationwide sample. Anesthesiology **2010** Apr;112(4):917-25.

4. Angus DC, Linde-Zwirble WT, Lidicker J, Clermont G, Carcillo J, Pinsky MR. Epidemiology of severe sepsis in the United States: analysis of incidence, outcome, and associated costs of care. Crit Care Med **2001** Jul;29(7):1303-10.

5. Wang HE, Shapiro NI, Angus DC, Yealy DM. National estimates of severe sepsis in United States emergency departments. Crit Care Med **2007** Aug;35(8):1928-36.

6. Gustot T, Felleiter P, Pickkers P, et al. Impact of infection on the prognosis of critically ill cirrhotic patients: results from a large worldwide study. Liver Int **2014** Nov;34(10):1496-503.
